# Evidence-based clinical practice guidelines for management of colorectal polyps

**DOI:** 10.1007/s00535-021-01776-1

**Published:** 2021-03-12

**Authors:** Shinji Tanaka, Yusuke Saitoh, Takahisa Matsuda, Masahiro Igarashi, Takayuki Matsumoto, Yasushi Iwao, Yasumoto Suzuki, Ryoichi Nozaki, Tamotsu Sugai, Shiro Oka, Michio Itabashi, Ken-ichi Sugihara, Osamu Tsuruta, Ichiro Hirata, Hiroshi Nishida, Hiroto Miwa, Nobuyuki Enomoto, Tooru Shimosegawa, Kazuhiko Koike

**Affiliations:** 1 Guidelines Committee for Creating and Evaluating the “Evidence-Based Clinical Practice Guidelines for Management of Colorectal Polyps”, the Japanese Society of Gastroenterology (JSGE), 6F Shimbashi i-MARK Bldg., 2-6-2 Shimbashi, Minato-ku, Tokyo, 105-0004 Japan; 2 Department of Endoscopy, Hiroshima University Hospital, 1-2-3 Minami-ku, KasumiHiroshima, 734-8551 Japan

**Keywords:** Colorectal polyp, Colorectal tumor, Polyposis, Treatment, Minds guidelines

## Abstract

**Background:**

The Japanese Society of Gastroenterology (JSGE) published ‘‘Daicho Polyp Shinryo Guideline 2014′’ in Japanese and a part of this guideline was published in English as “Evidence-based clinical practice guidelines for management of colorectal polyps” in the *Journal of Gastroenterology* in 2015. A revised version of the Japanese-language guideline was published in 2020, and here we introduce a part of the contents of revised version.

**Methods:**

The guideline committee discussed and drew up a series of clinical questions (CQs). Recommendation statements for the CQs were limited to items with multiple therapeutic options. Items with established conclusions that had 100% agreement with previous guidelines (background questions) and items with no (or old) evidence that are topics for future research (future research questions: FRQs) were given descriptions only. To address the CQs and FRQs, PubMed, ICHUSHI, and other sources were searched for relevant articles published in English from 1983 to October 2018 and articles published in Japanese from 1983 to November 2018. The Japan Medical Library Association was also commissioned to search for relevant materials. Manual searches were performed for questions with insufficient online references.

**Results:**

The professional committee created 18 CQs and statements concerning the current concept and diagnosis/treatment of various colorectal polyps, including their epidemiology, screening, pathophysiology, definition and classification, diagnosis, management, practical treatment, complications, and surveillance after treatment, and other colorectal lesions (submucosal tumors, nonneoplastic polyps, polyposis, hereditary tumors, ulcerative colitis-associated tumors/carcinomas).

**Conclusions:**

After evaluation by the moderators, evidence-based clinical practice guidelines for management of colorectal polyps were proposed for 2020. This report addresses the therapeutic related CQs introduced when formulating these guidelines.

## Introduction

The morbidity and mortality rates associated with colorectal cancer have increased in Japan with increasing westernization of diet and an aging society. The colon has become a prominent factor in health during the twenty-first century. Against this background, the Japanese Society of Gastroenterology (JSGE) published the second edition of ‘‘Daicho Polyp Shinryo Guidelines’’ (PG) in 2020 [1] following publication of the English version of the first edition [2]. Although the title indicates “colorectal polyps,” the PG covers all lesions located in the colon, including superficial and other neoplastic lesions, early-stage cancers, and polyposis.

In preparation for drafting the PG, we first set up an editorial committee and an evaluation committee. The committee members were recommended by the Japanese Gastroenterological Association, Japanese Society of Gastrointestinal Cancer Screening, Japanese Gastroenterological Endoscopy Society, Japan Society of Coloproctology, and Japanese Society for Cancer of the Colon and Rectum. To create the PG, the editorial committee met face-to-face and communicated by e-mail to discuss and draw up clinical questions (CQ). Recommendation statements for the CQ were limited to items with multiple therapeutic options. Items with established conclusions that had 100% agreement with previous guidelines (background questions) and items with no (or old) evidence that are topics for future research (future research questions: FRQs) were given descriptions only. Next, the evaluation committee was asked to examine and approve the questions to be included in the drafted CQs. A literature search formula was created for each CQ. PubMed, ICHUSHI, and other sources searched for relevant English-language articles from 1983 to October 2018 and for Japanese-language articles from 1983 to November 2018 for CQs and FRQs. The Japan Medical Library Association was also commissioned to search for relevant materials. Manual searches were performed for questions with insufficient online references. Finally, we created a structured abstract and completed the statements and descriptions. The recommendation grades and evidence levels were determined by the editorial committee following deliberations using the Delphi method. A draft of the guidelines was examined by the evaluation committee and then released to members of the participating societies for comment. The responses were discussed to finalize the PG. The PG contains sections on epidemiology, screening, pathophysiology, definition/classification, diagnosis, treatment and management, complications, and post-treatment surveillance, and includes other colorectal lesions (submucosal tumors, non-neoplastic polyps, polyposis, hereditary tumors, ulcerative colitis-associated tumors/cancers). The PG were created by incorporating the ideas in the Minds Handbook for Clinical Practice Guideline Development 2014 [3] and the JSGE clinical practice guidelines [4].

Finally, although the PG is to be used by general clinicians treating colorectal lesions; it is ultimately only a standard reference and should be used in conjunction with careful consideration of each patient's preferences, age, complications, and social situation. This paper introduces the CQs for the treatment of colorectal polyps including the surveillance after treatment underlying the contents of the PG.

## Clinical questions and statements

### CQ. What are the indications for endoscopic resection with respect to the size and shape of adenomas?

- Endoscopic resection should be performed for lesions ≥ 6 mm in size (recommendation strong [agreement rate 100%], level of evidence B).
- Endoscopic resection should also be performed in principle for diminutive polypoid adenomas ≤ 5 mm in size; however, follow-up observation by colonoscopy is also acceptable (recommendation weak [agreement rate 82%], level of evidence D).
- Endoscopic resection should be performed for flat and depressed neoplastic lesions even if ≤ 5 mm in size (recommendation strong [agreement rate 100%], level of evidence D).

Comment: In line with the widely accepted findings of the US National Polyp Study that colonoscopic removal of precancerous adenomas can prevent 76–90% of the morbidity associated with colorectal cancer and reduce the mortality rate by 53% [5, 6], total colonoscopy (TCS) and endoscopic resection of neoplastic lesions is now widely performed in Japan.

It is strongly recommended that endoscopic resection be performed for lesions ≥ 6 mm in size because the incidence of carcinoma is higher in lesions ≥ 6 mm than in those ≤ 5 mm (taking the relative risk of carcinoma for lesions ≤ 5 mm as 1, it increases to 7.2, 12.7, and 14.6 for lesions measuring 6–10 mm, 11–20 mm, and 20 mm, respectively) [7] and because it is often difficult to distinguish between benign adenoma and carcinoma by colonoscopy alone [7–9].

Based on the results of meta-analyses, endoscopic polypectomy [7] and endoscopic mucosal resection (EMR) [10, 11]/endoscopic submucosal dissection (ESD) [12] are now the preferred as optimal, less invasive treatments for colorectal neoplasia [13, 14]. However, 70–80% of all polyps detected in routine practice are diminutive (≤ 5 mm) [15], and the incidence of carcinoma in diminutive colorectal lesions in Western countries is reported to be exceedingly rare, ranging from 0.03% to 0.3% [9, 16].

Regarding the natural history of diminutive colorectal lesions, Western researchers detected recurrences of advanced lesions (invasive cancers, intramucosal cancers, adenomas measuring ≥ 10 mm, and villous or tubulovillous lesions) by follow-up colonoscopy in 4.2% of cases within 3 years after complete removal of all lesions [17]. Furthermore, a meta-analysis reported that 6% of 1,034 lesions measuring 1–9 mm in diameter progressed to advanced lesions within 2–3 years [18]. By contrast, a prospective cohort study of diminutive colorectal lesions in Japan found little change in either the size or shape of lesions after 2–3 years of follow-up and concluded that follow-up by colonoscopy without endoscopic resection is acceptable for these lesions [19, 20].

However, the AGA/ESGE guidelines strongly recommend endoscopic resection of all detected adenomas regardless of their size [21, 22]. There have also been reports that raise doubt about the ability of endoscopic removal of diminutive colorectal adenomas to prevent colon cancer [15, 16].

There is still controversy in Japan regarding whether or not diminutive adenomas should be removed, but after application of the Delphi method by the members of the working committee, it is weakly recommended in principle that all detected colorectal adenomas, including those that are diminutive, be endoscopically resected to prevent progression to cancer. However, depending on the patient's age, general condition, comorbidities, and personal wishes, follow-up observation by colonoscopy is acceptable for diminutive polypoid-type adenomas.

On the other hand, flat and depressed neoplastic lesions and those suspected of being carcinoma on colonoscopy are preferably treated by EMR regardless of size [23]. Colonoscopic findings suspicious for carcinoma include (1) expansive appearance (protrusion and overextension of the lesion and/or surrounding normal mucosa, such as a submucosal tumor [SMT]); (2) a depressed surface; (3) rough appearance (rough surface without shine); (4) a sessile elevation in the depressed area; and (5) a type V pit pattern (irregular or disappearance of surface structure). Chromoendoscopy or magnifying colonoscopy is recommended to confirm these findings [24, 25].

### CQ. How should hyperplastic polyps be managed?

- Follow-up is recommended for hyperplastic polyps ≤ 5 mm detected in the rectosigmoid region (recommendation weak [agreement rate 100%], level of evidence B).

Comment: Hyperplastic polyp (HP) is categorized as a serrated colorectal lesion along with sessile serrated adenoma/polyp (SSA/P) and traditional serrated adenoma (TSA) [26, 27].

Hyperplastic polyps presenting as whitish flat lesions ≤ 5 mm in size in the rectosigmoid region should be followed up because (1) there have been no reports on the association of these lesions with adenoma [28] and (2) there have been reports on precise endoscopic diagnosis of these lesions with combined use of dye spraying and/or narrow-band imaging (NBI) [29]. This statement is also recommended in the guidelines of the AGA/ESGE [21, 22].

### CQ. What are the indications for cold snare polypectomy?

- Cold snare polypectomy (CSP) is indicated for non-pedunculated benign adenomas < 10 mm in size (recommendation weak [agreement rate 100%], level of evidence B).
- CSP is recommended for diminutive lesions ≤ 5 mm in size and is acceptable for 6–9-mm lesions (recommendation strong [agreement rate 100%], level of evidence B).
- CSP should be avoided for “flat and depressed-type” lesions and lesions suspected of being carcinoma on colonoscopy even if ≤ 5 mm in size (recommendation weak [agreement rate 100%], level of evidence B).

Comment: CSP was developed by Tappero et al. in 1992 as an endoscopic resection technique for diminutive colorectal lesions ≤ 5 mm in size [30]. Since the lesion is mechanically resected without electrocautery, the incidence rates of post-polypectomy bleeding, post-polypectomy syndrome (peritonitis due to thermal injury), and delayed perforation are low with a shorter procedure and resection time. Due to these advantages, CSP is now widely used in Japan. CSP can be used for non-pedunculated benign adenomas < 10 mm in size. For diminutive lesions ≤ 5 mm in size, CSP is a superior resection method with a higher complete resection rate and a shorter resection time than conventional snare polypectomy or cold forceps polypectomy [31, 32].

According to the ESGE guidelines, CSP is strongly recommended as a good resection method for diminutive lesions ≤ 5 mm in size but weakly recommended for lesions 6–9 mm in size [22]. Furthermore, a Japanese randomized trial [33], a meta-analysis [34], and a histological evaluation [35] concluded that CSP could be a standard endoscopic resection method for 6–9-mm lesions. The complete resection rate is reported to be higher when using dedicated snare compared with a conventional snare [36, 37]. Cold forceps polypectomy using biopsy forceps is applicable for lesions up to 3 mm in size [22].

For lesions measuring ≥ 6 mm, however, CSP has some disadvantages compared with conventional polypectomy or EMR: there is a higher bleeding risk [38], about 10% of lesions cannot be retrieved, the muscularis mucosa resection rate is low, and the amount of resectable submucosa is limited. When CSP is performed for a lesion ≥ 6 mm in size, operators should be aware of the low histologically complete resection rate even for intramucosal lesions (incomplete resection rate, 3.9% [95% confidence interval 1.7–6.1]; unclear lateral margin rate, 67.1%) [39, 40]. Furthermore, caution is advised when CSP is used for lesions in the right colon, especially those in the cecum, and for SSA/P, in view of the low complete resection rate [41].

Moreover, due to the difficulty of performing a detailed histological evaluation of the smooth muscle (SM) layer in specimens resected by CSP, CSP should not be used for lesions with the possibility of SM invasion, such as when cancer is strongly suspected, or for “flat and depressed-type” lesions even when they are ≤ 5 mm in size [41]. It has been reported that lesion < 10 mm in size can be safely resected by CSP (using a dedicated snare) in patients taking antithrombotic agents [37, 42–44].

### CQ. How should serrated colorectal lesions be diagnosed and treated?

- Serrated lesions of the colorectum include SSA/P, TSA, and HP. The localization of the lesion, its form, and observation of the surface features (including magnifying endoscopic findings) are important for endoscopic diagnosis. SSA/P and TSA have the potential to develop into cancer, so treatment is recommended (recommendation weak [agreement rate 100%], level of evidence C).

Comment: Endoscopic diagnosis of serrated colorectal lesions is made by conventional observation and by image-enhanced endoscopic observation and in terms of the pit pattern with magnifying observation.

An SSA/P typically occurs in the right colon and is often a flat or broad-based lesion that is greater than 10 mm in diameter, pale in color, and associated with large amounts of mucin on the surface of the lesion [45]. NBI and magnifying observation of an SSA/P shows Japan NBI Expert Team (JNET) type 1 [46]. The dilated and branching (varicose microvascular) vessels reported by Uraoka et al. [47] are useful findings for diagnosis of SSA/P. A type II-open pit is characterized according to the findings on magnifying observation [48]. Co-existence of dysplasia and/or cancer is suspected in a lesion associated with central depression, reddishness, and a two-tier raised appearance [49]. Furthermore, type II-open and other pit patterns, including a mixture of type III, IV, and V pits, are found in most SSA/P with dysplasia and/or cancer [48, 50]. It has also been reported that co-existence of a cancer can be diagnosed when an irregular blood vessel can be seen on NBI with magnifying observation [51].

A TSA is a protruding lesion with distinct redness that is commonly found in the left side of the colon and rectum. The surface structure characteristically presents with a "pinecone" [52] or "branch coral-like” appearance [53]. TSA has distinctive endoscopic findings, making a diagnosis possible. A capillary network that expands widely into the stroma is observed on NBI [54].

The magnifying endoscopic findings for TSA are reported as IIIH pit (fernlike), IVH pit [55], and saw type IV (pit with the lobe-like findings of the fern) [56]. TSA can thus be distinguished from SSA/P and HP based on the endoscopic findings.

HP lesions have been reported in all segments, with predominance in the distal colon and rectum. They are commonly < 5 mm in size, pale in color and flat, and similar in circumference to the mucosa. JNET type 1 is seen on NBI and a type II pit pattern is seen on magnifying endoscopy.

SSA/P and TSA may undergo malignant transformation and therefore should be treated. An SSA/P is associated with BRAF mutations and the CpG island methylator phenotype and is considered a precursor lesion of colorectal cancer with microsatellite instability [57]. Recent studies have reported that SSA/P progresses to cancer in 1.5%–20% of cases [58]. Endoscopic resection should be performed for SSA/P [59].

Histologically, TSA can potentially progress to cancer. Therefore, treatment is indicated. As with typical adenomas, resection is advised for TSA > 5 mm in diameter. Most studies have recommended that SSA/P lesions > 10 mm in diameter be resected [60–62]. However, when co-existence of dysplasia and/or cancer is suspected, serrated lesions should be removed regardless of size [63].

HP may be a precursor lesion of SSA/P and/or TSA; however, treatment is not indicated for HP < 5 mm in diameter.

### CQ. What therapy is indicated for laterally spreading tumors?

- The choice of treatment between ESD and piecemeal EMR for a large laterally spreading tumor (LST) should be based on the subtype of LST with use of magnifying endoscopy and endoscopic ultrasonography as appropriate (recommendation strong [agreement rate 100%], level of evidence C).

Comment: LSTs are classified according to morphology into granular type (LST-G) and non-granular type (LST-NG) [64]. LST-G can be further subdivided into a ‘‘homogenous type’’ and a ‘‘nodular mixed type’’ and LST-NG into a ‘‘flat elevated type’’ and a ‘‘pseudo-depressed type’’. Most LST-Gs are considered adenomatous lesions. The incidence of carcinoma or submucosal invasion in LST-Gs is extremely low [65, 66]. A large nodule in a nodular mixed-type LST-G, where submucosal invasion tends to be present [66], should be resected en bloc [66, 67]. An adenomatous LST-G of the homogenous type can be resected by piecemeal EMR [67, 68]. A flat elevated-type LST-NG should be treated according to the preoperative diagnosis. For pseudo-depressed LST-NGs, en bloc resection should be performed, given that these tumors have a high probability of multifocal submucosal invasion independent of their size or pit pattern [67, 68]. In summary, the indications for ESD or piecemeal EMR are based on the LST subtype. Magnifying endoscopy and endoscopic ultrasonography can also be used as needed.

### CQ. In which colorectal tumors is it acceptable to perform piecemeal EMR?

- Definite adenoma or Tis carcinoma based on preoperative diagnosis are acceptable for piecemeal EMR. However, local recurrence rates are high with piecemeal resection and caution is therefore advised (recommendation weak [agreement rate 100%], level of evidence C).

Comment: In principle, en bloc resection should be performed for suspicious or definite cancer because the specimen obtained by complete en bloc resection needs to be examined pathologically in detail. Based on precise preoperative diagnosis with magnifying endoscopy, adenomatous lesions or focal carcinoma in adenomas > 2 cm in diameter, for which en bloc snare EMR is difficult, can be completely resected using deliberate piecemeal EMR to avoid segmentation of the carcinomatous area without compromising the pathological diagnosis [69]. Although the local recurrence rate associated with piecemeal resection is higher than that after en bloc resection [67, 70–73], most locally recurrent lesions are adenomas. Cure is possible with additional endoscopic treatment for local recurrent intramucosal lesions [67, 70–73]. In contrast, ESD allows complete en bloc resection regardless of lesion size. However, colorectal ESD is technically more difficult and requires considerable experience.

### CQ. How should surveillance colonoscopy be planned after endoscopic removal of colorectal adenoma?

- Follow-up colonoscopy should be performed within 3 years after polypectomy. (recommendation weak [agreement rate 100%], level of evidence B).

Comment: The US National Polyp Study Workgroup recommended an interval of at least 3 years between complete colonoscopic removal of adenomatous polyps and postoperative follow-up examination [74]. Furthermore, based on the initial TCS findings, a 5-year cumulative incidence of advanced neoplasia (large adenoma ≥ 10 mm, villous tumor, high-grade dysplasia, or cancer) was reported after removal. Based on this result, the significance of the baseline TCS findings was established (Table 1) [75]. According to the European guidelines [76] and modified US guidelines [21], the most suitable interval for surveillance colonoscopy is recommended based on the number of adenomas, maximum size of the polyps, and histopathological findings (including the presence of villous components or high-grade dysplasia) of the resected lesions. As a general guideline, patients with three or more adenomatous polyps < 10 mm (low-grade dysplasia [LGD]) or polyps with high-grade dysplasia or villous components should undergo surveillance colonoscopy at 3 years post-polypectomy. In contrast, patients with only one or two small low-grade dysplastic lesions should undergo routine screening (i.e., a fecal occult blood test) at 10 years post-excision according to the European guidelines and surveillance colonoscopy after 5–10 years according to the US guidelines. Moreover, detailed risk stratification from baseline TCS findings, such as those with large numbers (≥ 10) of adenomatous polyps or sessile serrated lesions can help define individual recommended TCS intervals. In Japan, the decision to follow these guidelines is not determined because management of diminutive adenoma (< 5 mm) has not been established. In brief, endoscopists in the West attempt to remove all adenomatous polyps whereas there is no uniform Japanese approach (to removal or follow-up) for diminutive adenomas. Therefore, this topic remains controversial in Japan [77–81]. The present guidelines recommend the following based on data from a retrospective study by the Japan Polyp Study Workgroup [82]: ‘‘Follow-up colonoscopy should be performed within 3 years after polypectomy.’’.

**Table 1:** Relative risk of advanced neoplasia within 5.5 years based on findings at baseline

Baseline findings (*n*, with examination)	No advanced neoplasia, *n* (%)	Advanced neoplasia, *n* (%)	Relative risk	95% CI
No neoplasia (298)	291 (97.6)	7 (2.4)	1.00	
Tubular adenoma < 10 mm (622)	584 (93.9)	38 (6.1)	2.56	1.16–5.67
1 or 2 (496)	473 (95.4)	23 (4.6)	1.92	0.83–4.42
> 3 (126)	111 (88.1)	15 (11.9)	5.01	2.10–11.96
Tubular adenoma > 10 mm (123)	104 (84.6)	19 (15.5)	6.40	2.74–14.94
Villous adenoma (81)	68 (83.9)	13 (16.1)	6.05	2.48–14.71
High-grade dysplasia (46)	38 (82.6)	8 (17.4)	6.87	2.61–18.07
Cancer (23)	15 (65.2)	8 (34.8)	13.56	5.54–33.18

*CI* confidence interval. Adapted from Lieberman et al. [76]

### CQ. How should surveillance be planned after endoscopic resection of T1 (SM) colorectal cancer?

● Close monitoring is necessary for not only local recurrence but also lymph node metastasis and distant metastasis. Careful follow-up for a minimum of 3 years should be performed after endoscopic resection (recommendation weak [agreement rate 100%], level of evidence C).

Comment: Based on the results of a multicenter retrospective cohort study, additional surgery with lymph node dissection should be recommended from a perspective of “long-term recurrence risk,” even if one of the factors in “simultaneous lymph node metastasis risk” is positive [83].

1. Endoscopic resection alone (low-risk) group: patients who were treated by endoscopic resection because their evaluation satisfied the curative criteria in the colorectal cancer treatment (JSCCR) guidelines [84].
2. Endoscopic resection alone (high-risk) group: patients who did not meet the curative criteria for endoscopic resection but were followed up without surgery.
3. Endoscopic resection + surgery (high-risk) group: patients who did not meet the curative criteria for endoscopic resection and underwent additional surgery.
4. Surgery group: Patients who underwent surgery as the initial treatment.

This retrospective study was conducted with a focus on the metachronous recurrence rate of 626 cases of T1 (SM) colorectal carcinoma in which endoscopic resection was selected as the initial treatment. A recurrence rate of 7.1% was observed in the endoscopic resection alone (high-risk) group in which additional surgery was not performed deviating from the guidelines. However, the recurrence rate was 1.9% and the 5-year recurrence-free survival rate was 98% in the endoscopic resection alone (low-risk) group, which was a better result than that in the group that underwent endoscopic resection alone (Table 2). Furthermore, distant recurrence (liver, lung, or bone metastasis) was observed in 10 of 17 cases in this study. We concur with the recommendations in other reports that close monitoring is needed not only for local recurrence but also lymph node metastasis and distant metastasis when following up without additional surgery despite deviating from the curative criteria in the guidelines. Careful follow-up for at least 3 years is required (there are rare reports of recurrences after 3 years, and a 5-year follow-up is preferable if possible) [85–89]. Furthermore, it should be noted that the recurrence rate is higher in T1 (SM) rectal cancer than in colon cancer [90]. Currently, there is no clear recommendation regarding a surveillance strategy after endoscopic resection for T1 (SM) colorectal cancer. The minimum evaluation should include measurement of tumor markers (CEA/CA19-9) at 6-monthly intervals, chest and abdominal computed tomography (CT; chest and pelvis CT in the case of rectal cancer) and colonoscopy (first follow-up after 6 months and annually thereafter).

**Table 2:** Long-term outcomes according to treatment/risk group

Group	Cases, * n*	Recurrence, * n* (%)	5-year recurrence-free survival, %	5-year overall survival, %
ER alone (low-risk)	105	2 (1.9)	98	93
ER alone (high-risk)	84	6 (7.1)	90	97
ER + surgery (high-risk)	159	4 (2.5)	97	98
Surgery	278	5 (1.4)	98	99

*ER* endoscopic resection. Adapted from Yoda et al. [85]

Moreover, not only the rate of synchronous lymph node metastasis but also the metachronous recurrence rate is much lower than that of non-pedunculated lesions, in the case of pedunculated T1 (SM) carcinoma. Therefore, if head invasion alone is detected, lymphovascular invasion is negative, and tumor budding is grade 1, then follow-up with endoscopic resection only can be considered [91, 92].

### CQ. What is the diagnosis and management of neuroendocrine tumors of the colorectum?

- When a rectal SMT is detected, especially in the lower rectum, a neuroendocrine tumor (NET) is the most likely possibility. The strong recommendation is to confirm that the tumor surface is covered with normal mucosa using the dye spray method and whether the tumor needs to be resected by endoscopy or surgery depending on its size and surface characteristics (recommendation strong [agreement rate 100%], level of evidence B).

Comments: According to the 2019 World Health Organization classification [93], the generic term for pancreatic and gastrointestinal tumors with endocrine properties and phenotypes is neuroendocrine neoplasm. These tumors are classified into well-differentiated NET and poorly differentiated neuroendocrine carcinoma (NEC). NET is subdivided into G1, G2, and G3 based on cell proliferating activity. NET G3, which had been classified as NEC in the fourth edition of the classification has since been reclassified as a type of NET based on results of clinical and genetic studies. Moreover, in the fifth edition, mixed neuroendocrine-non neuroendocrine neoplasm is a new category that was previously classified as mixed adenoneuroendocrine carcinoma.

The type of tumor previously known as “carcinoid tumor” corresponds to NET G1 and G2. The difference between NET G1 and G2 is based on the Ki-67-positive cell expression rate, which is calculated by counting the number of Ki-67-positive cells in 1,000 tumor cells where Ki-67 positive cells most commonly occur. The tumor is classified as G2 if the Ki-67-positive cell expression rate is ≥ 3% and as G1 if the rate is < 3%.

## Diagnosis of colorectal NET

Rectal NET accounts for 99% of all NETs in the large intestine, and about 80% are located within 10 cm of the dentate line. NET appears as a yellowish SMT because it arises from the deep lamina propria layer of the mucosa and grows into the submucosal layer at an early stage. When an SMT is detected, especially if in the lower rectum, NET is the most likely diagnosis. It should be confirmed that its surface is covered with normal mucosa by the dye spray method [94, 95]. If NET is suspected, concomitant use of endoscopic ultrasonography is recommended. NET is usually visualized by endoscopic ultrasonography as a uniform hypoechoic mass [94–96].

## Treatment of colorectal NET

Before treatment of colorectal NET, CT or magnetic resonance imaging should be performed to confirm that there is no distant or lymph node metastasis. Management of NET according to size is described in the following sections [96–98].

1. Less than 10 mm in diameter
  Endoscopic resection is recommended when no depressed surface or ulcer is observed on the tumor surface and the tumor confined to T1 (SM). The preferred resection methods are EMR, EMR-L method (EMR using a ligation device) [99, 100], EMR by the cap method [101] or ESD [102]. According to a meta-analysis, the EMR-L, cap, and ESD methods had a higher complete resection rate than conventional EMR [103]. Post-resected specimens are evaluated to identify risk factors for lymph node metastasis, which will be described later, and to determine whether additional treatment is required [104, 105]. The prognosis is reported to be good after endoscopic resection of lesions of this size [106].
2. 10 mm or more in diameter.
  In principle, given that the incidence of lymph node metastasis has been reported to increase to 18.7%–30.4% for lesions ≥ 10 mm in diameter [96–98], surgical resection with lymph node dissection should be performed for lesions of this size. However, depending on the patient's age, general condition, comorbidities, and personal wishes, complete resection by local excision as a complete excisional biopsy is acceptable if the lesion is confined to T1 (SM). After assessing the risk factors for lymph node metastasis in the resected specimen, which will be described later [104, 105], the need for additional treatment can be considered.

Clinicopathological risk factors for lymph node metastasis in rectal NET include the following: (1) tumor diameter ≥ 11 mm; (2) positive depressed surface/ulcer on the tumor surface; (3) T2 (MP) or deeper invasion than T2; (4) positive lymphatic permeation; (5) 2 or more mitoses/10 high-power fields (visual field × 400); and (6) a Ki67 labeling index ≤ 3% (NET G2) [93, 96–98, 104, 105].

## Follow-up after resection of rectal NET

Surveillance after resection of rectal NET should be the same as for colorectal cancer. Given the slow growth of NET, longer-term follow-up is required [107]. It has been reported that positron emission tomography/CT using 68 Ga-DOTA-peptide is useful for evaluating the treatment effect for primary and metastatic colorectal NET [108].

### CQ. How should non-neoplastic polyps be managed?

- We recommend classifying non-neoplastic colorectal polyps as hamartomatous, inflammatory, or hyperplastic (recommendation strong [agreement rate 100%], level of evidence D).
- While most non-neoplastic colorectal polyps are not indicated for endoscopic removal, we recommend removal of symptomatic polyps if they are a source of bleeding, cause intussusception, or are suspected of being cancer (recommendation strong [agreement rate 100%], level of evidence D).

Comments: Non-neoplastic colorectal polyps are histologically classified as hamartomatous (e.g., Peutz-Jeghers polyp, juvenile polyp), inflammatory (or benign lymphofollicular), or hyperplastic [109]. A non-neoplastic polyp can be easily distinguished from an adenomatous polyp based on endoscopic findings, including macroscopic configuration, color, surface architecture, and pit pattern. However, endoscopic biopsy is inevitable for polyps with an obscure diagnosis [110].

Due to the lower rate of malignant transformation when compared to adenomatous polyps, observation seems to be appropriate for most non-neoplastic colorectal polyps. However, endoscopic or surgical resection is indicated for polyps that cause clinical symptoms, such as bleeding or intussusception, and those with presumably malignant transformation.

### CQ. Are there differences in management between FAP and attenuated FAP?

- Surveillance colonoscopy from adolescence onward is recommended for both familial adenomatous polyposis (FAP) and attenuated FAP (AFAP) (recommendation strong [agreement rate 100%], level of evidence C).
- Prophylactic proctocolectomy is recommended for AFAP because it complicates colorectal cancer (recommendation strong [agreement rate 100%], level of evidence C).

Comments: AFAP is defined as the presence of up to 100 colorectal adenomas in subjects aged 25 years or older [111]. FAP includes both APC and MUTYH variants and there is a high rate of colorectal cancer in each subtype [112–114]. Therefore, surveillance colonoscopy starting at a younger age is necessary for AFAP. Even though there have been reports of patients with AFAP remaining free of colorectal cancer throughout their lives [115], the high risk of colorectal cancer in these patients suggests a need for prophylactic proctocolectomy [111, 116, 117]. Total colectomy with ileorectal anastomosis may be appropriate in AFAP because the incidence of cancer in the rectal remnant is lower in AFAP than in FAP. However, lifelong surveillance for colorectal cancer is mandatory in patients with AFAP. In contrast, total proctocolectomy with ileal-pouch-anal anastomosis is recommended for FAP [118]. There have been clinical trials of non-surgical interventions involving tight endoscopic removal in patients with FAP and AFAP who have refused surgery, but the long-term efficacy of this strategy remains to be elucidated [119].

### CQ. Who are candidates and what are appropriate methods for cancer surveillance in ulcerative colitis?

- We recommend that initial surveillance colonoscopy be performed in patients with extensive left-sided colitis that is present 8–10 years after onset of the disease(recommendation strong [agreement rate 100%], level of evidence B).
- We suggest starting surveillance earlier for patients in whom remission is not confirmed endoscopically (recommendation weak [agreement rate 100%], level of evidence B).
- We recommend total colonoscopy using chromoendoscopy and/or NBI with targeted biopsy. Attention should be paid to elevated lesions and any change in mucosal structure or color that is different from that in the surrounding area (recommendation strong [agreement rate 100%], level of evidence B).
- We recommend stepped biopsies from each segment of the colon (recommendation weak [agreement rate 100%], level of evidence B).

Comments: Endoscopic surveillance for cancer in patients with ulcerative colitis has been shown to be effective in reducing colorectal cancer-related mortality [120]. The guidelines recommend surveillance colonoscopy starting 8–10 years after disease onset [121, 122]. Given that dysplastic lesions are difficult to detect in colonic mucosa with active inflammation, surveillance endoscopy should be performed during periods of endoscopic remission to the extent possible.

Individualization of the surveillance interval based on stratification of the risk of colorectal cancer is recommended. According to the guidelines published by the British Society of Gastroenterology and Association of Coloproctology of Great Britain and Ireland [123], patients should be stratified as follows: (1) lower risk (extensive colitis with no endoscopic/histological active inflammation or left-sided colitis); (2) intermediate risk (extensive colitis with mild active endoscopic/histological inflammation or presence of post-inflammatory polyps or family history of colorectal cancer in a first-degree relative aged 50 years or over); and (3) higher risk (extensive colitis with moderate/severe active endoscopic/histological inflammation or stricture in the past 5 years, dysplasia in the past 5 years, or primary sclerosing cholangitis or a family history of colorectal cancer in a first-degree relative aged younger than 50 years). Colonoscopy is recommended at 5-year intervals for the lower risk group, 3-year intervals for the intermediate risk group, and annually for the higher risk group. According to the European Crohn’s and Colitis Organisation guidelines [122], the next surveillance colonoscopy should be scheduled for 1 year in higher risk patients (with severe active inflammation, inflammatory polyps, strictures, or LGD detected), 2–3 years in intermediate risk patients (with persistent mild inflammation), and 5 years in lower risk patients.

Chromoendoscopy has been shown to improve the dysplasia detection rate [123–125]. However, the effectiveness of NBI for surveillance remains controversial [125, 126].

Although it has been shown that a targeted biopsy is as effective as a conventional random biopsy for cancer surveillance [127], there are lesions that are difficult to detect endoscopically. The targeted biopsy in addition to stepped (random) biopsy should be used on a case-by-case basis. The guidelines recommend that stepped biopsy specimens should be taken from each segment of the colon [120, 121].

### CQ. If dysplasia or cancer is detected in ulcerative colitis, should be all lesions be surgical removed? Is low-grade dysplasia an indication for surgery?

- If LGD is detected in flat mucosa, we suggest a consultation with several experienced pathologists (recommendation weak [agreement rate 100%], level of evidence C).
- If LGD is detected in an elevated lesion and sporadic adenoma is highly probable, the recommendation is to perform endoscopic resection and a detailed pathological examination (recommendation strong [agreement rate 100%], level of evidence C).
- However, total proctocolectomy is recommended if cancer or high-grade dysplasia is found and determined to be colitis-associated (recommendation strong [agreement rate 100%], level of evidence C).

Comments: At present, there is no consensus on the treatment strategy for lesions diagnosed as LGD in flat mucosa. Some follow-up studies have found frequent progression to high-grade dysplasia and cancer in cases diagnosed as LGD [128–130] whereas others have reported that progression is unlikely [131, 132]. A recent meta-analysis reported that the incidence of progression to colorectal cancer was 0.8% [133]. Given the interobserver variability in the diagnosis of dysplasia, LGD detected in flat mucosa should be confirmed by several pathologists with expertise in inflammatory bowel disease. The follow-up interval should be determined in consultation with expert gastroenterologists and surgeons.

Differentiation between colitis-associated dysplastic lesions and sporadic adenomas is very important. Endoscopic resection should be performed for lesions that are highly likely to be sporadic adenomas and a detailed histopathological examination by an experienced pathologist is recommended [122, 125]. However, because elevated lesions diagnosed as dysplasia may have high-grade atypia in the deep layer even if the surface atypia is low-grade, or may be accompanied by invasive cancer, intensive endoscopic surveillance should be performed after about 3 months. Furthermore, endoscopic resection is an option for lesions outside the affected area that are likely to be sporadic adenoma [122, 125].

## Appendix

Members of the working committee who created and evaluated the ‘‘Evidence-based clinical guidelines for management of colorectal polyps’’, JSGE.

## Executive Committee

Chair: Shinji Tanaka (Department of Endoscopy, Hiroshima University Hospital).

Vice-Chair: Yusuke Saitoh (Digestive Disease Center, Asahikawa City Hospital).

Members: Takahisa Matsuda (Endoscopy Division, National Cancer Center.

Hospital), Takayuki Matsumoto (Division of Gastroenterology, Iwate Medical.

University), Yasushi Iwao (Center for Preventive Medicine, Keio University.

Hospital), Masahiro Igarashi (Department of Endoscopy, Cancer Institute Ariake.

Hospital), Yasumoto Suzuki (Coloproctology Center, Matsushima Clinic),

Tamotsu Sugai (Department of Molecular Diagnostic Pathology, Iwate Medical.

University), Michio Itabashi (Department of Surgery, Tokyo Women’s Medical.

University), Ryoichi Nozaki (Coloproctology Center Takano Hospital), Shiro Oka.

(Department of Gastroenterology and Metabolism, Hiroshima University Hospital).

## Evaluation Committee

Chair: Ken-ichi Sugihara (Department of Surgical Oncology, Tokyo Medical and Dental University).

Vice-Chair: Osamu Tsuruta (Department of Gastroenterology, St. Mary's Hospital).

Members: Ichiro Hirata (Department of Gastroenterology, Osaka Central Hospital), Hiroshi Nishida (Occupational Health Center, Panasonic Health Insurance Organization).

## The Japanese Society of Gastroenterology

President: Kazuhiko Koike (Department of Gastroenterology, the University of Tokyo).

Past President: Tooru Shimosegawa (South Miyagi Medical Center).

Director Responsible: Hiroto Miwa (Division of Gastroenterology, Department of Internal Medicine, Hyogo College of Medicine), Nobuyuki Enomoto (First Department of Internal Medicine, Faculty of Medicine, University of Yamanashi).
